# A Systematic Review: Assessment of the Metabolomic Profile and Anti-Nutritional Factors of *Cannabis sativa* as a Feed Additive for Ruminants

**DOI:** 10.3390/metabo14120712

**Published:** 2024-12-18

**Authors:** Tumisho Ntsoane, Ndivho Nemukondeni, Lufuno Ethel Nemadodzi

**Affiliations:** 1 Department of Agriculture and Animal Health, University of South Africa, Science Campus, Florida 1709, South Africa; 58547444@mylife.unisa.ac.za; 2 Department of Animal Sciences, Tshwane University of Technology, Pretoria 0001, South Africa; nemukondenin@tut.ac.za

**Keywords:** *Cannabis sativa*, anti-nutritional factors, fermentation, nutritional value, metabolomic profile, legislature

## Abstract

**Background:** *Cannabis sativa* is a high-value crop that can be cultivated for ruminant’s feed and medicinal purposes. The demand for Cannabis and Cannabis products has increased since the beginning of 21st century. **Objectives:** The increase in the production cost of high-protein feeds such as lucerne has led to an urgent need to investigate alternative high-protein sources. **Methods**: Cannabis has been identified as an alternative to lucerne due to its high protein content. **Results**: However, the cultivation and uses of Cannabis and its by-products in South Africa is limited due to the strict legislation. The metabolites and nutritional value of Cannabis are influenced by growing conditions and soil type. Furthermore, the available literature has shown that Cannabis contains anti-nutritional factors that may affect feed intake or bioavailability and digestibility. **Conclusions:** Therefore, it is crucial to employ a processing method that can reduce anti-nutritional factors to promote the feed intake and growth rate of sheep. Fermentation, as a processing method, can reduce anti-nutritional factors found in Cannabis, which will make it a palatable alternative feed supplement for ruminants such as Dorper sheep. Overall, this review paper aimed to examine the available literature on the use of Cannabis as an alternative high-protein feed supplement for Dorper sheep in South Africa.

## 1. Introduction

**Figure 1:**
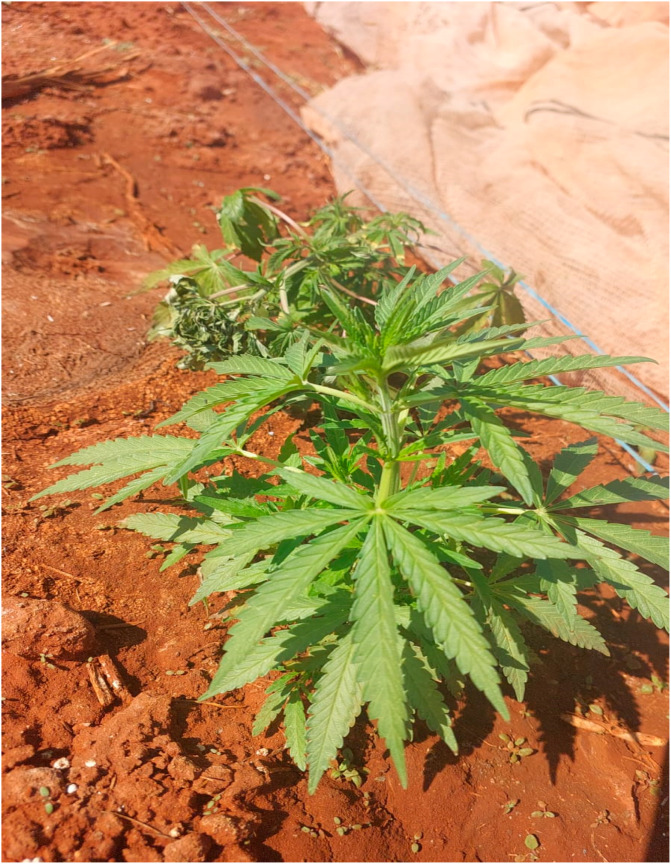
Cannabis plant at the flowering stage grown at Towoomba Research station (Ntsoane T., July 2024).

*Cannabis sativa*, commonly known as marijuana (Figure 1), is a herbaceous flowering annual plant that belongs to the family of *Cannabaceae* and the genus *Cannabis* [1,2]. Cannabis is a well-known name that is used to identify other various strains and subspecies such as *Cannabis ruderalis* and *Cannabis indica* [3]. In African countries such as Nigeria, Cannabis is commonly known as Gbάnά in Yoruba, while in the Democratic Republic of Congo, in the Lingala language, it is known as Diamba. In South Africa, in Limpopo Province, it is known as Lebake and Mošwang wa matuba by the Bapedi tribe, and Mbanzhe by the VhaVenda tribe, whilst the Vatsonga tribe refers to it as Imbange. Additionally, in Kwa-Zulu Natal, the AmaZulu tribe calls it Izolo.

**Figure 2:**
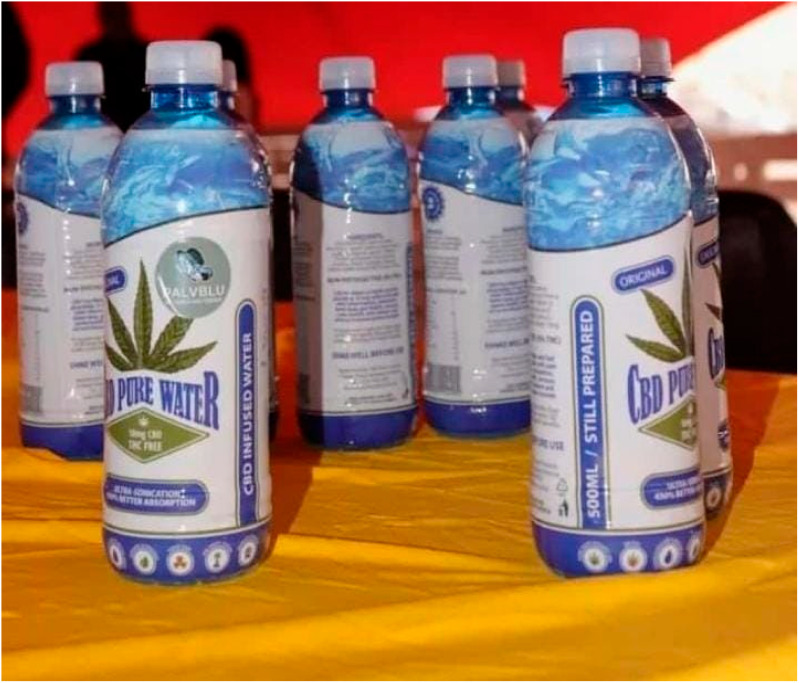
Bottled Cannabis-infused water (Matsena S.D., July 2023).

Previous studies have indicated that there are over 600 strains of Cannabis that are available commercially [4]. Cannabis has been used in medicine as well as as a source of textile fiber since it was discovered [5]. This is one of the crops that is most exploited and misused mainly for recreational purposes [6]; as a result, it has negative implications associated with it, which have reduced efforts to expand research and development. However, in South Africa, the traditional uses of Cannabis include boiling the leaves and steam to cure flu, as well as drinking Cannabis water (Figure 2) to strengthen the immune system. Furthermore, several studies have reported that medication that contains metabolites such as cannabinoids may be beneficial for the treatment of certain rare kinds of epilepsy, nausea, and vomiting caused by cancer chemotherapy, as well as lack of appetite, and weight loss caused by HIV/AIDS [7,8,9].

Planting Cannabis without permission is still restricted in South Africa; however, in 2018, the constitutional court ruled in favour of the Cannabis for Private Purpose Bill, which allows people to plant Cannabis for private use [10]. The excellent medicinal properties of Cannabis together with the relaxation of legislation [6] regarding its use in many parts of the world, such as China [11], the United States of America [12], as well as South Africa [6], in the past few years, have motivated researchers and farmers to explore other possibilities and uses of the Cannabis plant for industrial purposes and as a feed supplement for ruminants. However, the use of Cannabis as a fodder crop is often limited by legal restrictions related mostly to its cultivation, as well as concerns over its potential psychoactive effects on animals. In South Africa, the limitations regarding industrial Cannabis are mainly focused on cultivation rather than phytochemicals that may be present in animal products such as meat and milk destined for human consumption. Legislative limitations reduce the availability of Cannabis as a protein supplement to farmers, as they can only plant a limited number of plants when they do not have legal permission.

**Figure 3:**
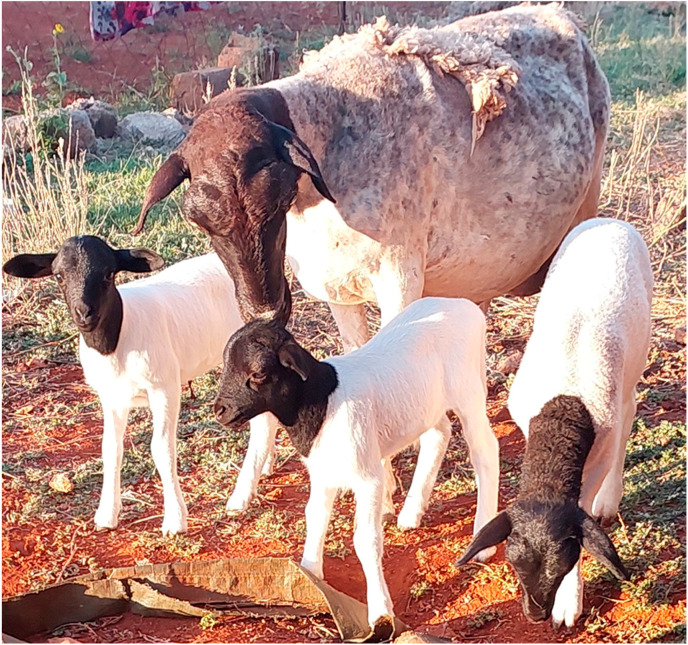
Dorper sheep raising 3 lambs at GaMphahlele, Limpopo Province (Ntsoane T., March 2022).

The use of ruminants such as sheep has remained a substantial and versatile livelihood strategy for the majority of rural and resource-disadvantaged people in South Africa [13]. Smallholder farmers encounter several limiting factors that emphasize the need to farm with a sheep breed that has a higher feed conversion ratio (FCR) and good growth rate [14]. Dorper sheep (Figure 3) are a hardy hybrid breed that originated in South Africa as a cross between Blackhead Persian ewes and Dorset Horn rams. It is regarded as an early maturing, hardy, and fertile breed that often weans multiple lambs with minimum human intervention [15]. It has been observed and reported that Dorper sheep have a higher growth rate compared to indigenous breeds such as Bapedi and Damara sheep, which are also found in South Africa (unpublished). Over the past decade, smallholder farmers have changed their flocks from indigenous breeds to Dorper sheep [16], aiming to benefit from their good growth rate and fertility, thus increasing household income. Dorper sheep’s grazing habits are non-selective; they utilize shrubs and bushes with available grasses on natural vegetation [17]. To date, no studies have been conducted in South Africa on Cannabis inclusion in the diet of Dorper sheep; therefore, future studies are required to explore the use of Cannabis as part of the Dorper sheep’s diet.

**Figure 4:**
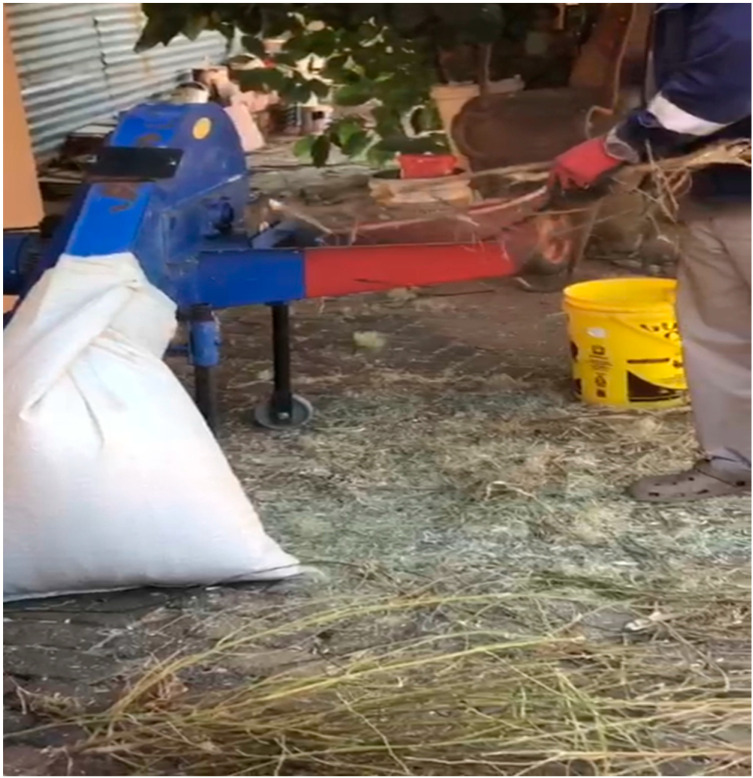
Grinding sun-dried Cannabis for ruminants’ feeds with a hammer mill in Limpopo Province, Mankweng municipality (Matsena S.D., July 2023).

A recent study by Rehman et al. [18] revealed that Cannabis has been used as a ruminant feed supplement in different parts of the world, such as in Asian, American, and European countries. The cultivation of this medicinal plant has been carried out for a thousand years; its seeds, leaves, and stems have been utilized by numerous herbivores, particularly ruminants such as cattle, goats, and sheep, across different parts of the world [19], as well as by poultry [20]. Farmers have been using Cannabis as a feed for ruminants (Figure 4) in rural parts of South Africa; however, they are doing so without any formal and scientific guidelines, and they rely on their indigenous knowledge. Sun-dried Cannabis leaves, stems, and in some cases the inflorescence are crushed with a grinding machine, which is then fed to animals such as cattle, goats, pigs, and sheep as a protein supplement.

Constant increases in the prices of commercial livestock protein sources and their lack of accessibility due to distance have led to the need to use alternative high-protein fodder sources such as Cannabis for the partial or total replacement of commercial livestock protein sources by smallholder farmers, as shown in Figure 4. Commercial high-protein fodder crops such as lucerne (*Madicago sativa*), also known as alfalfa, and soybean (*Glycine max*) are resource-intensive in terms of crop management practices [21], and their input costs have increased significantly in the last few years. Cannabis is known to produce large quantities of biomass within a short period that can sustain and provide adequate daily nutritional needs for ruminants [22]. When ruminants get adequate daily nutritional input, they grow and reproduce well, thus increasing the household income for smallholder farmers.

Due to the novelty of Cannabis as a revived crop in some parts of the world, such as the United States of America [23] and South Africa, the information on the levels of Cannabis inclusion in ruminant feeding and the effects of anti-nutritional factors on the intake of Cannabis feed by Dorper sheep is limited.

**Figure 5:**
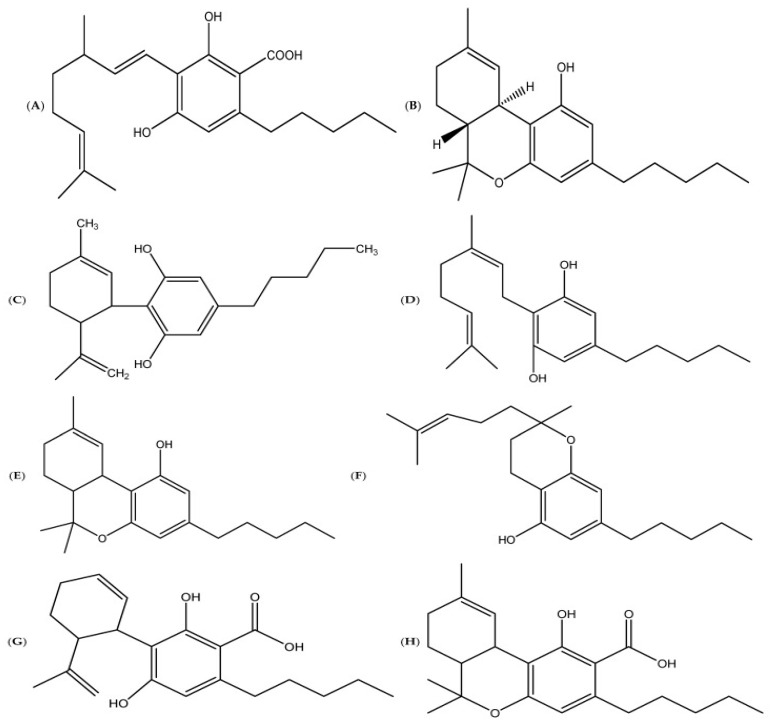
Chemical structures of the most common cannabinoids, including (**A**) cannabigerolic acid (CBGA); (**B**) delta-9-tetrahydrocannabinol (Δ9-THC); (**C**) cannabidiol (CBD); (**D**) cannabigerol (CBG); (**E**) cannabinol (CBN); (**F**) cannabichromene (CBC); (**G**) cannabidiolic acid (CBDA); and (**H**) tetrahydrocannabinolic acid A (THCA-A) [6].

The nutritional value of Cannabis makes it ideal for ruminants as it provides a rich source of proteins [24], essential fatty acids [5], vitamins [19], and minerals [25]. Moreover, these proteins and fatty acids are important for the growth and brain function of ruminants [26]. Additionally, Cannabis contains different natural phytochemical compounds (Figure 5) such as tetrahydrocannabinol (Δ*^9^*-THC) [27], cannabidiol (CBD) [28], and other metabolites [29]. In addition to Δ*^9^*-THC and CBD, there are four other substantial compounds found in *C. sativa,* which are tetrahydrocannabivarin (THCV), cannabinol (CBN), cannabigerol (CBG), and cannabichromene (CBC) [30]. Furthermore, a recent study by Milay et al. [31] indicated that there are over 500 compounds reported from different varieties of Cannabis, including *C. sativa*. Cannabis grown in different parts of the world may synthesize different levels and types of metabolites due to differences in the environmental conditions, soil types, and the genotypes of the plant [29].

Metabolomics has been widely used in various human and plant investigations [32]; however, there is a scarcity of information available on the metabolomics profile of *C. sativa* and other varieties of Cannabis in South Africa. Additionally, Monyela et al. [6] indicated that fewer research studies have been aimed at uncovering new phytochemicals of various Cannabis strains. The study of metabolomics provides us with the ability to study small molecules within samples so as to understand the underlying influence of interaction between genetics, the environment, and stressors [33]. The historical restrictions on Cannabis throughout the world have delayed the ability to conduct the necessary research required to comprehend its value [34]. In South Africa, the studies relating to the metabolomic profiles of various crops and soils are gaining momentum; even so, the metabolomic profiles of various parts of Cannabis other than seeds and flowers and the influences of planting season on metabolomic profiles are still limited. To overcome limitations, significant and in-depth research is required.

Cannabis has been subjected to crossbreeding between its three distinct varieties over the years; therefore, it has become a complex plant species that produces a vast array of bioactive phytochemicals [6]. The characterization of the hundreds of compounds found in Cannabis will provide a difficult challenge for standard analytical chemistry, but one that is necessary for the emerging discipline of metabolomics [4]. However, compound characterization is often limited to a few categories that are mostly based on the plant’s cannabinoid content. In South Africa, there are also limited research studies aimed at the discovery of new phytochemicals of various Cannabis strains [6] that may possess anti-nutritional factors and metabolites that may reduce the intake of Cannabis by ruminants. The findings of studies on the metabolomic profile of Cannabis conducted in countries such as Canada and the United States of America cannot be extrapolated and used as a point of reference in the South African context due to differences in soil types and environmental factors. Yang et al. [35] indicated that external environmental factors such as photoperiod, temperature, and soil fertility affect processes associated with the growth and development of plants and their ability to accumulate and synthesize secondary metabolites, all of which eventually leads to an overall change in the metabolomic profile of the plant.

Cannabis seeds have good nutritional value [36]. Studies conducted by Klir et al. [3] indicated that Cannabis seeds may be included in the diets of ruminants, as they provide a valuable source of protein and fatty acids without significant changes in the production traits of animals that utilize them. Additionally, Cannabis seeds have about 20 to 25% protein content of high biological value, which is easily digestible and rich in essential amino acids [19]. Recent studies by Bailoni et al., Axentii and Codină [24,37] revealed that Cannabis contains anti-nutritional factors such as tannins, saponins, phytic acid, trypsin inhibitors, and cyanogenic glycosides, which may affect the intake and bioavailability of feed and subsequently lead to the poor growth performance of ruminants. There is no published literature available on the use of the whole plant or different botanical parts such as leaves and stems as parts of the diets of ruminants in South Africa. More studies need to be conducted to give a better understanding of the anti-nutritional factors of the whole Cannabis plant. Ultimately, broader research will assist with the development of guidelines and regulations for Cannabis inclusion in the ruminant diet.

The anti-nutritional factors found in Cannabis may need to be reduced to promote the palatability of Cannabis to ruminants, as well as to increase the digestibility and bioavailability of nutrients to ruminants. A reliable and cost-effective processing method such as fermentation should be employed, as suggested by Adebiyi et al. [38]. Fermentation is required to reduce and/or prevent the loss of nutrients present in plant-based animal feed ingredients [39]. Currently, there are no studies that have been conducted on the fermentation of Cannabis to reduce feed loss and promote livestock productivity, or to provide guidelines on appropriate fermentation periods to reduce the anti-nutritional factors of Cannabis planted in South Africa. Therefore, the purpose of this review is to investigate the available scientific literature on the uses and benefits of Cannabis as a supplementary source of diet for ruminants, thereby creating awareness among livestock farmers.

## 2. Materials and Methods

**Figure 6:**
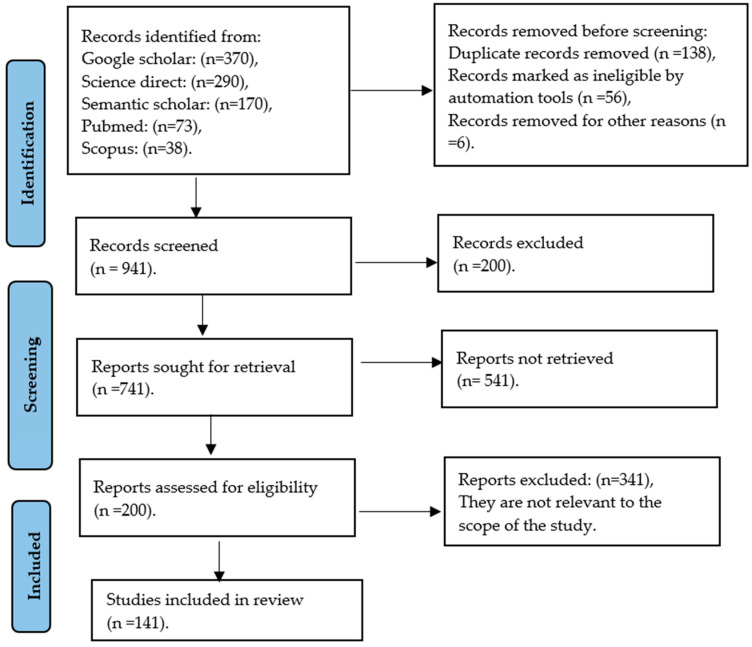
Identification of studies via databases and registers; the diagram shows the methods by which relevant studies were retrieved from the databases and assessed, selected, or excluded.

Searches of relevant topics, such as metabolomics profile and anti-nutritional factors of Cannabis, were used to produce a systematic review. PRISMA (Preferred Reporting Items for Systematic Reviews and Meta-Analyses) was followed as a guide to complete this systematic review [40], and the full search is outlined in Figure 6 below.

### 2.1. Search Strategies

A search was carried out to generate a systematic literature base by examining five scientific databases, including Science Direct, Google Scholar, Semantic Scholar, PubMed, and Scopus. The following words were used in combination: “*Cannabis sativa*”, “metabolomic profile”, “anti-nutritional factors”, “fermentation”, “growth performance”, “legislation”, “Dorper sheep”, and “Lucerne”. The year of publication was used as a limitation to retrieve articles, review papers, and research reports published in English only, from the period 2014–2024.

### 2.2. Selection Criteria

The selection criteria were based on the PRISMA statement [40]. The search focused on original research papers published between 2014 and 2024 that studied Cannabis plants, anti-nutritional factors, and the inclusion of Cannabis in feeds. Cannabis plants were not limited by their botanical components (leaves, stems, and inflorescence) and herbal form (flowering tops, or hash oil), variety (*C. indica*, *C. sativa*, or *C. ruderalis*), or gender (male, female, or monoecious). Articles published before 2014 were excluded from the search.

### 2.3. Risk of Bias Assessment

The study relied completely on original research articles, review papers, and conference papers. To ensure the quality of the review, all duplications were extensively scrutinized. The review method included article abstracts to guarantee the academic literature’s quality and relevance. Each research report was carefully evaluated at a later stage.

## 3. Results and Discussion

### 3.1. The Use of Metabolomics to Advance Cannabis Research Subsection

Metabolomics study is crucial in Cannabinoid profiling as it helps to identify and quantify different types of Cannabinoids such as THC, CBD, and CBG [41]. Metabolomics studies involve the identification and classification of all tiny molecules, generally known as metabolites, in a biological system [42]. It refers to the overall study of metabolite expression at any given time [43,44,45], and it is rapidly expanding in agriculture and biology, focusing on measuring and describing the profiles of all metabolites found in soil, plant or animal extracts [46]. Additionally, according to a recent study conducted by Jadhav et al. [47], metabolomics provides clarity on biological systems as they develop and respond to changes in the environment, and provides clearer insight when used together with genomics, transcriptomics, and proteomics. Moreover, metabolomics has proven to be an effective and advanced research tool that provides a clearer link to the phenotype of an organism, and helps solve difficult research questions that could not be solved by conventional methods. However, in Africa, particularly South Africa, the use of metabolomics over conventional methods to advance Cannabis research is still lagging. Consequently, future research studies should include metabolomics to examine metabolites that are yet to be explored, in order to advance not only Cannabis research, but also research into other important crops.

**Figure 7:**
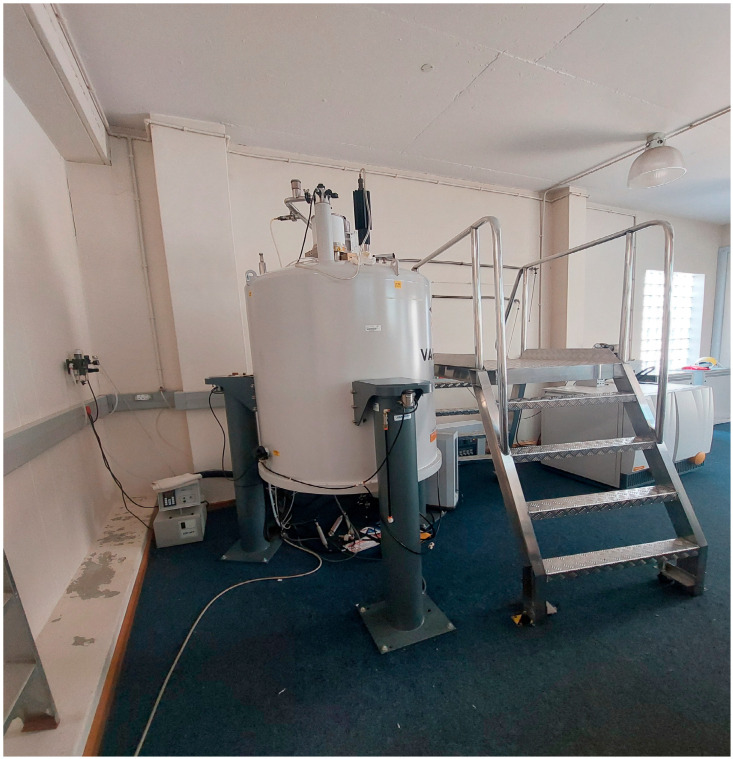
Nuclear magnetic resonance (NMR) 600 MHz spectroscopy, CSIR, Pretoria (Ntsoane T., 6 May 2024).

Metabolomics utilizes modern computational methods and high-throughput analytical chemistry tools to identify and characterize hundreds of compounds in complex biochemical combinations [48]. It is generally accomplished by using qualitative techniques, which include nuclear magnetic resonance (NMR) spectroscopy (Figure 7) and mass spectrometry (MS) in conjunction with a high-resolution separation technique such as liquid (LC) or gas chromatography (GC) [49], and these ultimately enable the detection of many metabolites in a single sample. The purpose of metabolomics is to track changes in the concentrations of a wide number of metabolic markers throughout time [50]. A recent study by Zhong et al. [51] indicated that metabolomic studies frequently employ radiolabeled nutrients or other inputs to quantitatively compare distinct profiles. Numerous plant species, including Cannabis, will benefit from the successful application of metabolomics, and the amount of data produced by this technique related to Cannabis will increase exponentially in the future [52]. Additionally, the data obtained from the study of metabolites found in cannabis plants are important in developing the plant’s potency, uses, benefits, and efficacy. Moreover, analyzing the metabolomic profiles of different cannabis strains can assist with plant breeding programs, wherein strains that have desirable traits and that may be included in a ruminant diet can be developed. Research on the metabolomics of Cannabis in Africa is lagging, except for in a few plant and soil studies conducted in South Africa [6].

A recent study conducted by Colellea at al. [53] indicated that NMR spectroscopy can provide valuable quantitative and qualitative information on the chemical composition of Cannabis seed and flower extracts, which illustrates that NMR spectroscopy is a useful tool in characterizing the targeted metabolites present in the Cannabis plant. There are two major compounds found in *C. sativa*, which are cannabinoids and non-cannabinoids; the non-cannabinoids include phenolics, flavonoids, terpenes, as well as alkaloids [30]. In addition, according to Rodríguez-Daza et al. [54], phenolics can be distinguished into flavonoids and non-flavonoids, with the latter representing phenolic acids, stilbenes, tannins, and coumarins. A study by Iffland et al. and Sarma et al. [55,56] indicated that some Cannabinoids such as THC can impact ruminants negatively due to their potential to cause lethargy, confusion, and loss of coordination. However, a recent study by Hassan et al. [57] showed some of the Cannabinoids, such as CBD, may have a positive impact on ruminants, such as reduction in stress and inflammation. Furthermore, a study conducted by Samuel [58] showed that the inclusion of Cannabis, particularly varieties with higher CBD content, may promote fertility in cows, which will result in higher income for farmers. Additionally, according to Yang et al. [59], other Cannabinoids such as CBG and CBC, have antimicrobial and anti-inflammation effects, which may improve gut health as well as feed efficiency in ruminants. Nonetheless, little research has been performed on the toxicity of THC and other Cannabinoids for ruminants in South Africa. Future research should investigate the effects of Cannabinoids on ruminants, which will assist with the development of regulations and guidelines on the use of Cannabis as part of ruminant nutrition in South Africa.

A recent study by Bassolino et al. [60] reported that the inflorescence of *C. sativa,* particularly from a female plant, contains plenty of secondary metabolites; furthermore, secondary metabolites such as Cannabinoids and terpenes have been studied more widely than non-Cannabinoids, including terpenes, flavonoids and alkaloids, which may influence ruminant nutrition [61,62,63]. Terpenes such as limonene, pinene and myrcene, among others [64], and flavonoids such as quercetin and kaempferol may have numerous positive impacts on ruminants, such as antimicrobial properties, anti-inflammatory effects, as well as antioxidant properties, which improve the gut health, feed efficiency and respiratory health of ruminants [65]. Additionally, alkaloids such as choline have a positive impact on ruminants, as they help with ruminant liver health and fat metabolism [66], while alkaloids such as trigonelline help with glucose metabolism and insulin sensitivity in ruminants [67], which is essential in maintaining glucose homeostasis. In South Africa, there is limited scientific literature available; as a result, more research that involves in vivo studies should be conducted to determine the impacts of metabolites found in *C. sativa* on the nutrition of ruminants.

There are about 125 cannabinoids, of which 5 have been discovered in the past two years, with 42 phenolics, 34 flavonoids, 120 terpenes, and 2 alkaloids identified in *C. sativa* [30]. Additionally, the available literature shows that the most abundant flavonoids found in *C. sativa* are flavones and flavonols [60,68,69]. Furthermore, Bassolino et al. [60] studied the synthesis of phenols in different *C. sativa* varieties, and their findings reported 211 compounds; it was also indicated that the pattern of phenolic compounds and accumulation is influenced by the genotype. Moreover, studies conducted by Micalizzi et al. [70] indicated there are about 150 cannabinoids present in *C. sativa*, and the discrepancy may be due to different varieties of *C. sativa*, growing conditions, and other factors that are known to affect the metabolomic profile of *C. sativa*. Due to the variance of growing conditions affecting Cannabis throughout the world, it is important to further investigate the impacts of the growing conditions and genetic makeup, as well as the other botanical components other than seeds and inflorescence, of *Cannabis sativa,* on its metabolomic profile in South Africa. Studies on botanical components other than Cannabis seed and flower are still lacking. Future studies must investigate the metabolites found in botanical components of cannabis to uncover additional metabolites and the underlying nutritional potential and benefits of the Cannabis plant. Additionally, future work must be expanded on adding new metabolites to the database; currently, signals observed in untargeted metabolites research still cannot be detected because their spectra are not available in databases [71].

In South Africa, *C. sativa* has been given a number of names; however, according to [72], there is a lack of documentation on Cannabis varieties in Southern Africa. Additionally, the National Centre for Biotechnology Information (NCBI) has identified and assembled 15 genomes of Cannabis [72]; nonetheless, these samples do not represent the samples that are in the hands of South Africans because their genotypes are different. Future studies must explore the knowledge gap surrounding genetic diversity and metabolomic profiles of Cannabis varieties found in South African Cannabis. It is important to establish a reference genome for local Cannabis varieties to significantly advance Cannabis research and development in South Africa. These advancements will eventually benefit the local community while also advancing the global understanding of the crop.

### 3.2. Nutritional Value of Cannabis

The nutritional values of many different crops are affected by many factors, which include the type of crop, the variety, the growth stage, the available soil nutrients, the amount of rainfall, and the management of the crop [73]. A recent study by Mekasha et al. [74] showed that it is apparent that the time of the year, location, variety, and seeding rates affect biomass dry matter accumulation and nutritional value of various crops, including Cannabis. Cannabis is grown at a commercial or industrial level for its botanical components, as shown in Table 1, except for the roots [22]. For instance, the lucerne and Cannabis stem had the lowest crude protein contents compared to Cannabis leaves and seeds. The three botanical parts of Cannabis had the highest crude fiber contents compared to lucerne. Cannabis seed had lower acid detergent fiber and neutral detergent fiber. Overall, Cannabis has a better nutritional composition compared to lucerne, which makes it an ideal source of protein for ruminants. The values indicate the nutritional composition of Cannabis, and the health benefits Cannabis can offer to the livestock industry. However, the figures are derived from a limited number of available studies performed on Cannabis. To develop more concrete estimations, more studies should be conducted on the nutritional composition of Cannabis across all available varieties under different environmental conditions. However, the current systematic review paper focuses on the botanical parts grown for commercial purposes.

Several studies conducted by Cockson et al., Kleinhen et al., and Araiza-Rosales et al. [77,78,79] indicated that Cannabis contains 20–25% protein, which is higher than in the common high-protein fodder crops such as lucerne (see Table 1). Klir et al. [3] evaluated the possibility of using Cannabis in animal nutrition, and indicated that Cannabis contains a significant amount of fiber, vitamins, minerals, and essential fatty acids. Further studies still need to be done to determine the nutritional benefits and provide regulations and guidelines on the effective use of Cannabis as part of ruminant feed. Future studies should report on the influence of the phytochemical composition of Cannabis, such as tetrahydrocannabinol and cannabidiol, on livestock products such as meat and milk. Furthermore, future work should investigate the impact of THC on the palatability of livestock products, such that regulations can be developed on the acceptable amount of Cannabis that can be included in ruminant nutrition to ensure food security and safety.

### 3.3. Anti-Nutritional Factors of Cannabis Sativa

A study by Samtiya et al. [80] reported that some plant-based feed ingredients contain anti-nutritional factors, including *C. sativa*, which may affect ruminant nutrition negatively. For instance, Jin et al. [81] found 14 metabolites such as cannabidivarin (Cbdv), cannabidarinic acid (CBDVA), CBG, CBD, CBDA, THCV, CBGA, CBN, (−)-Delta9-THC-D3, (−)-Delta 9-THC, (−)-Delta8-THC, getrahydrocannabivarin (THCVA), CBC, THCA, and cannabichromenic acid (CBCA) in the stems, leaves and roots of *C. sativa*. The anti-nutritional factors are biological compounds that are found in various types of plant-based feed ingredients that reduce feed intake or bioavailability and metabolism [19,82]. Furthermore, they contribute to the impaired gastrointestinal and metabolic performance of animals upon feeding [19]. There are several anti-nutritional factors that are found in plant-based animal feed, which include saponins, phytic acid, trypsin inhibitors, cyanogenic glycosides, and tannins [83]. To date, no studies have been conducted on the use of botanical parts of Cannabis, such as leaves, roots, and stems, as animal feed. However, the available research focused mainly on the flowering part of Cannabis.

Phytic acid is a natural compound that serves as a main phosphorus storage form and the plant’s primary phosphorus reserve [19]. It is categorized as an anti-nutritional component in grain crops such as millet and legumes such as lucerne [84]. It can limit the bioavailability of nutrients; research has traditionally focused on its unique structure, which gives it the ability to bind minerals, proteins, and starch, and the resulting detrimental effects, such as decreasing the absorption of iron, zinc, magnesium, and calcium [85]. According to Galasso et al. [86], the phytic acid content in Cannabis seeds ranges between 4.5 and 7.6 g/100 g of the dry weight of the defatted matter, whilst Russo and Reggiani [87] reported a 6.2 to 7.7 g/100 g phytic acid content in Cannabis seed. Moreover, Mattila et al. [88] indicated that the phytic acid content in Cannabis seed is 5.3 g/100 g; these findings are in agreement with the findings made by Galasso et al. [86]. The variation in the results could be due to the influence of the environmental conditions, the genotype, and their interactions with the anti-nutritional factors. There is limited research that has been conducted on the concentration of phytic acid in the leaves, stems, and roots of Cannabis plants.

Condensed tannins (CT) are secondary plant polyphenol compounds that are reported to be anti-nutritional due to their capacity to bind protein in plant-based feeds, enzymes, and microbial cells [89]. As a result, CT interferes with microbial digestion and impedes ruminal protein and dry matter (DM) digestion, thus reducing the nutritional value and palatability of feeds [90]. However, condensed tannins are generally phenolic compounds, and can positively influence animal health due to their antioxidant capacity and anti-inflammatory properties related to the phenolic rings found in their structure [91].

Trypsin inhibitors (TIs) are one of the most relevant anti-nutritional factors that reduce the digestion and absorption of dietary proteins; they inhibit trypsin activity, which is the enzyme that breaks down proteins into smaller portions [92]. They are a form of endogenous anti-nutritional substance that is mostly discovered in numerous plants, seeds, and vegetables, including grains, nuts, oilseeds, and seeds from pulse and legume plants [93]. They affect the utilization of proteins and their digestion by both humans and animals, and may have a detrimental effect on the nutritional contents of food and feed products [94]. Trypsin inhibitors serve as the plant’s defence against microbial proteinases and as a regulator of endogenous proteinase activity [19]. Several studies conducted on the TIs of soybean and legume seeds, such as cowpea, have indicated that this content ranges between 10.8 and 28.4 U/mg [86,87,88]. The differences could be due to differences in the soil type and climatic conditions, as well as the genotype of Cannabis used in the research studies. Currently, the literature on the presence of TIs in Cannabis leaves is still lacking.

Cyanogenic glycosides are a significant and common family of plant natural products, which lack the structural variety of many other natural product classes [95]. They serve as defence agents against herbivores, in this case by releasing toxic hydrogen cyanide after tissue damage [19]. Some plant species are deadly for humans due to their high content of cyanogenic glycosides [96]. The level of cyanogenic glycosides produced by the plant depends on the age and variety of the plant, as well as the environmental conditions under which the plant grows [97]. According to recent findings by Huang [98], levels below 100 ppm of cyanogenic glycosides are not harmful to the health of animals, including humans. On the contrary, Russo and Reggiani [87] indicated that the contents of cyanogenic glycosides in Cannabis seeds range between 50 and 240 ppm, which are above the threshold of 100 ppm, which may be harmful to animals if consumed over an extended time. As a result, there is a need for further studies that will determine the correct processing methods (such as fermentation) to reduce cyanogenic glycosides. According to Huang et al. [98], cyanogenic glycosides can be reduced significantly after being subjected to 72 h of fermentation; these reductions make it safe to utilize the plant for ruminant feed. Future studies need to be performed to describe the minimum period for which it can be given to animals.

Saponins are a class of bioorganic compounds found in particular abundance in the plant kingdom [99]. They are naturally occurring bioorganic compounds with at least one glycosidic linkage (C-O-sugar bond) at C-3 between glycone and a sugar chain [100]. In larger quantities, these compounds are gastric irritants, and they can show toxic properties, causing hemolysis of the red blood cells [82]. However, at low doses, they may have a plasma cholesterol-lowering effect in animals including sheep, as well as strong cytotoxic effects against cancer cell lines, and are important in reducing the risk of many chronic diseases in humans [19]. A study by Russo and Reggiani [87] indicated that the content of saponins found in Cannabis seeds is low, and they have no harmful effects on ruminants fed Cannabis.

TIs and, most importantly, phytic acid are the most prevalent anti-nutritional chemicals among those examined in hempseed. Overall, it has been shown that all the examined anti-nutritional chemicals have positive correlations, which suggests that these compounds’ biosynthesis pathways in cannabis may be active during seed development [99]. The literature is scarce on anti-nutritional factors of other botanical parts of the Cannabis plant, which calls for future research on the evaluation of the effects of anti-nutritional factors on ruminants. Additionally, future research will strengthen regulations on the use of Cannabis inclusion in animal feeds and provide guidance on the safe use of feed containing anti-nutritional factors. A recent study by Anaemene and Fadupin [101] reported that fermentation improves nutritional value by reducing anti-nutrients within feeds or by enhancing health benefits by diversifying bioactive compounds. The abovementioned could be justified as fermentation reduces anti-nutritional factors, which could improve feed intake and digestibility, thereby increasing the growth rate of ruminants. In South Africa, the Department of Agriculture, Land Reform and Rural Development regulates anti-nutritional factors in animal feeds through the Fertilizers, Farm Feeds, Agricultural Remedies and Stock Remedies Act 24 of 1977. However, the Act does not provide guidelines and regulations on anti-nutritional factors found in Cannabis. Due to the limited research on and complexity of anti-nutritional factors, regulation agencies may lack resources for effective enforcement and compliance.

### 3.4. Fermentation as a Cost-Effective Processing Method to Reduce Anti-Nutritional Factors

Most plants commonly accepted as animal feed contain large amounts of nutrients needed by animals for growth and reproduction; however, they may also contain anti-nutritional factors that reduce nutrient bioavailability and palatability, which may affect the overall feed intake [82]. Anti-nutritional factors above the normal threshold are a great concern, as they reduce digestibility and metabolites, and may lead to nutrient and mineral deficiencies in ruminants in some cases. They may also affect the intake and digestibility of the ruminants, which may subsequently lead to their poor growth performance [102]. It therefore becomes very essential to reduce these anti-nutritional factors through reliable and cost-effective processing methods to make plant-based feed ingredients such as Cannabis more palatable and increase their intake and digestibility [103].

**Figure 8:**
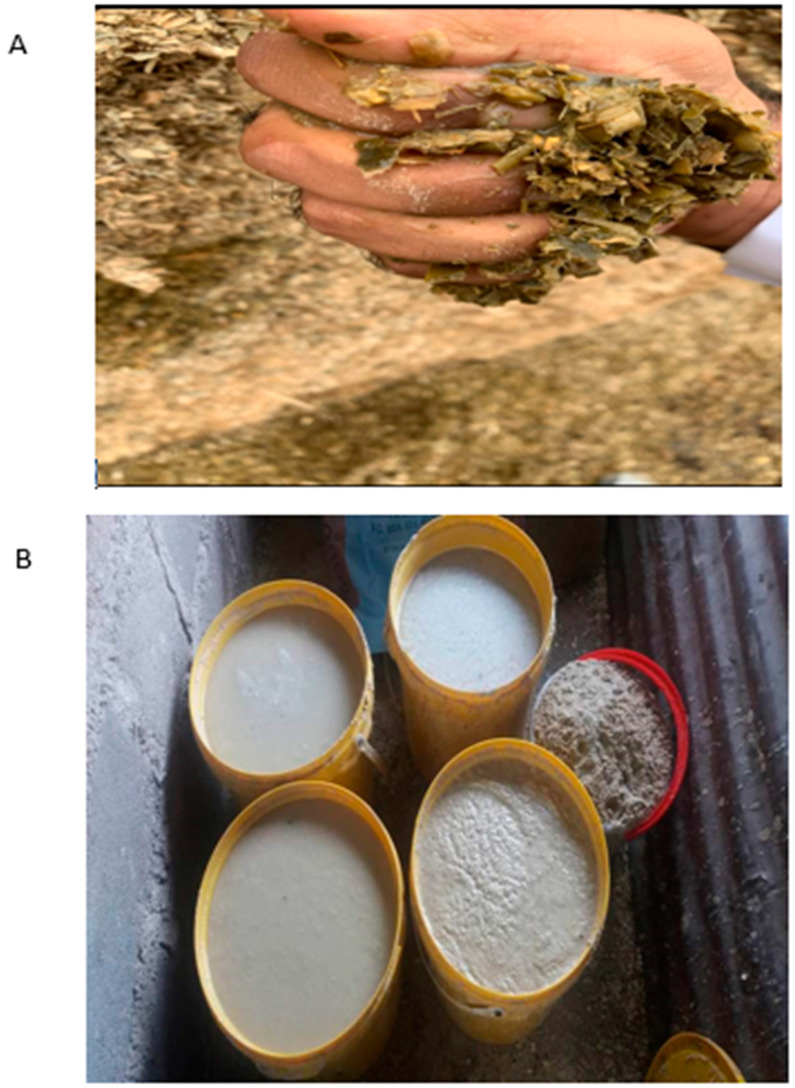
Silage anaerobic fermentation (**A**) and liquid fermentation (**B**) of commercial cattle feed, Tzaneen, South Africa (Matemane D., 22 June 2024).

Various processing techniques and processes, such as fermentation, germination, debranning, autoclaving, soaking, etc., may be used to reduce feed anti-nutritional factors concentrations [104]. It is feasible to lower the amount of anti-nutrients in ruminant feeds by utilizing a variety of approaches individually or in combination. One such possible processing method could be fermentation, particularly liquid fermentation, as suggested by Adebiyi et al. [38], as it is easily accessible and affordable even to smallholder livestock farmers. Common feed fermentation methods include solid state fermentation (SSF), which involves fermenting feed ingredients in the absence of free water [105], and liquid fermentation (LF), which includes fermenting feed ingredients in a liquid medium, as shown in Figure 8 [106]. The LF and SSF may be done under aerobic [107] and anaerobic [108] conditions, and bacteria such as *Lactobacillus plantarum* [109] and yeast [110] may be used. In South Africa, the LF method is mostly preferred by resource-poor smallholder farmers to ensure that ruminants absorb the available nutrients in the plant-based feed ingredients. The fermentation process takes about 48 to 72 h under anaerobic conditions before being fed to animals—mostly pigs and chickens, as well as ruminants. However, scientific studies should be conducted to determine and confirm the optimum hours required for the fermentation of active ingredients to ensure smooth and safe consumption by ruminants.

Fermentation is a metabolic process that oxidizes sugars to produce energy; it also increases mineral absorption from plant-based diets [111]. It is one of the processes used in Africa to make cereal crops palatable while improving the nutritional quality and safety of these meals, as cereals are not easily consumed in natural/raw forms [111]. This is an important method that considerably reduces the contents of anti-nutrients in grains such as phytic acid and tannins [112]. The reduction in anti-nutritional factors increases nutrient bioavailability, digestibility, and palatability in ruminants, and reduces pathogens in feed to enable an increased absorption of nutrients, and ultimately improve growth and performance [113].

Phytic acid generally forms complexes with metal cations such as iron, zinc, calcium, and proteins in some grains and legume crops [19]. Enzymes destroy these complexes, which require an optimal pH maintained by fermentation. As a result, this type of degradation can be reduced [114]. The phytic acid concentration is reduced, and soluble iron, zinc, and calcium are liberated, increasing the nutritional value of dietary grains [104]. The lactic acid bacterium (LAB) fermentation of cereals has been shown to increase free amino acids and their derivatives through proteolysis and metabolic synthesis [115]. Fermentation has been found to boost grain nutritional value by increasing the quantity of key amino acids such as lysine, methionine, and tryptophan [116].

Various studies [111,117,118] have shown that millet grain fermentation for 12, 24, and 36 h reduces the number of anti-nutritional factors, including protease inhibitors, phytic acids, and tannins. Fermentation also provides optimal pH conditions for the enzymatic breakdown of phytate, which is commonly found in grains in the form of complexes with polyvalent cations such as iron, zinc, calcium, magnesium, and proteins [117]. Additionally, a reduction in phytate may increase the quantity of soluble iron, zinc, and calcium by several orders of magnitude [119]. The reduction in anti-nutritional factors encourages an increase in essential minerals in the bodies of ruminants’, thus increasing absorption and promoting the growth rate and productivity of ruminants.

Tannin levels may be lowered by lactic acid fermentation, resulting in enhanced iron absorption, except in some high-tannin grains, where little or no improvement in iron availability has been documented [120]. A recent study by Samtiya et al. [80] reported that fermentation at 30 °C over 72 h resulted in an approximately 88.3% reduction in phytic acid content in most grain crops, such as millet and maize. Additionally, another study by Tanilas and Kriščiunaite [121] concluded that phytic acid levels were reduced to lower levels when rice flour was subjected to natural fermentation. According to Ogodo et al. [122], maize flour was fermented with a consortium of lactic acid bacteria by the standard method with 12 h intervals to check the effects of fermentation on anti-nutritional factors. These results show that fermentation reduces anti-nutritional factors significantly. Additionally, there are no studies that have investigated the effects of fermentation or the fermentation period on Cannabis.

The results by Yu et al. [123] suggest fermentation as one of the more cost-effective methods to reduce the anti-nutritional factors in feeds. Microbial fermentation activates many endogenous enzymes, such as phase, which generally degrades the phytate in the feed; phytate is one of the largest anti-nutritional factors, and is present in many crops, including Cannabis [111]. Previous works have established that anti-nutritionals have a close negative relationship with micronutrient bioavailability, because high contents of anti-nutritional factors reduce the bioavailability or absorption of minerals, and could lead to nutrient deficiency or malnutrition [122]. The anti-nutritional factors such as CT and secondary metabolites such as THC present in the Cannabis plant may bind to and reduce the availability of nutrients such as proteins essential for the growth of animals. Furthermore, a study by Vlassa et al. [111] reported that fermentation increased phenolic acids as well as crude protein. Another study by Xu et al. [124] reported that fermented feed ingredients positively affect ADG, and feed ratio (FR) elicits a growth-enhancing effect in weaners of pigs by increasing their nutrient availability and reducing their anti-nutritional factors. However, to date, no studies have been conducted on fermentation methods and their impacts on the anti-nutritional factors of Cannabis to be used as a supplement diet for Dorper sheep in South Africa. Fermentation using bacteria such as *Lactobacillus plantarum* [109], yeast [105], and solid-state fermentation [125] has proven to be effective in the reduction in anti-nutritional factors and in increasing the nutrient availability of plant-based feed ingredients such as mulberry, peanut meal, and rapeseed meal. Therefore, the quality of plant-based ingredients can be improved by subjection to fermentation, thus reducing the loss of nutrients and increasing the growth performance of ruminants. However, studies on the impacts of the fermentation of Cannabis, particularly its leaves and stems, are still lacking. Additionally, future research must explore the impacts of fermented Cannabis feed on the growth performance of ruminants such as Dorper sheep.

### 3.5. Inclusion of Cannabis Leaf in Ruminants Feed

In South Africa, lucerne is the common protein-rich feed supplement for ruminants; however, it has become expensive for most resource-poor farmers due to the increase in costs of agricultural inputs [126]. Climate change has resulted in changes in rainfall patterns; due to unpredictable and unreliable rainfalls, the establishment of lucerne is a challenge due to seedling vulnerability [127], as well as a lack of availability of machinery for soil preparation. Alternative affordable fodder sources are required to reduce feed shortage challenges during the dry season when there is limited feed available. Studies have found that Cannabis contains 20 to 25% protein [19], which content is higher than that of Lucerne, with a protein content of between 17 and 20% [34], thus making it a possible alternative fodder substitute for lucerne. Although there is limited literature on the use of Cannabis as a feed supplement for ruminants, particularly sheep, there have been reports that Cannabis leaves removed from stalks are fed to ruminants in some parts of the world, such as rural China [18]. More research still needs to be conducted in South Africa because the nutritive value of Cannabis may be affected by various factors such as variety, growth stage, soil fertility, available rainfall, and the management of the crop [74]. Consequently, research findings from America, Europe and Asian countries cannot be extrapolated to and used in South Africa.

Previous research found that farmers in Asian and European countries have been using Cannabis as ruminants feed, particularly for cattle and sheep [128]. The use of Cannabis seed as a whole, meal, cake, or oil for use in animal feed has been reported by several researchers [3,24,129]. Karlsson [130] reported that dairy cows fed with up to 31.8% Cannabis seed cake showed a significant increase in milk yield. Additionally, studies conducted by Ncogo Nchama et al. [131] indicated that the dry matter intake is not influenced by the addition of Cannabis seed in a feed meal fed to cattle. The findings illustrate the great advantage of the use of Cannabis as a supplement for ruminants, although studies on the inclusion of the use of other botanical parts of the Cannabis plant such as leaves, stems and roots are still lacking. Cannabis waste can be included in ruminant feed; even so, due to the presence of phytochemicals present in Cannabis, the feeding of Cannabis is still prohibited for animal products destined for human consumption in the USA [23]. Irawan et al. [132] reported the presence multiple cannabinoids in the liver tissues of lambs fed feed with spent hemp biomass (SHB). Furthermore, another study by Irawan et al. [133] indicated that feeding cows SHB decreased DMI, mainly due to the low palatability of the SHB pellet; however, a higher milk yield was obtained. However, in South Africa, there are no studies conducted on feeding ruminants with cannabis-based products, and no scientific literature is available.

#### Inclusion of Cannabis Meal as a Supplement to Sheep Diets

The growth performances of various types of ruminants such as sheep, goats, and cattle have been examined when fed with a meal that includes Cannabis seed [23]. A recent study conducted by Lourencon et al. [134] demonstrated that supplementing ruminants such as Dorper sheep results in an increase in body weight as well as body condition score. Additionally, Karlsson and Martinsson [135] conducted a study to evaluate the effects of the inclusion of Cannabis seed intake on sheep, and their findings agree with the results from Ncogo Nchama et al.’s [131] study, which revealed that the inclusion of Cannabis does not have an adverse effect on the dry matter intake. However, the findings on the average daily weight gain of ruminants from the same studies did not differ between meals that included Cannabis and a control treatment [133]; the similarities of the results might have been influenced by the fact that Cannabis seed meal was not subjected to fermentation to reduce the anti-nutritional factors present in the Cannabis seeds and to make nutrients available for the animals’ bodies to utilize. According to a recent study by Jacobson et al. [136], Cannabis can be used as an effective source of crude protein, as it contains sufficient protein to meet the minimum daily requirements of sheep. Moreover, the nutritional quality and biomass yield are affected by the photoperiod, which indicates that it is important to plant in the right planting season, when photoperiods are longer. Additionally, the photoperiod has an impact on metabolite synthesis [137]. Furthermore, a recent study by Lanzoni et al. [138] reported that the inclusion of Cannabis seeds in the diet of sows improved the nutritional quality of milk and colostrum in lactating sows, which led to the improved growth rate of piglets. However, the study does not recommend the amount of Cannabis that should be included in the diets of animals, or the duration of feeding. Kreb et al. [139] reported that the inclusion of Cannabis stubble in the pelleted diet of Merino sheep had no effect on dry matter (DM) intake, and there was a discrepancy in the results regarding the liveweight (LW) gain. Furthermore, Ran et al. [140] agreed with the abovementioned findings, that substituting lucerne hay with cannabis did not cause an increase in the body weight or average daily gain (ADG) of goats; additionally, dry matter intake decreased with increasing substitution rates of Cannabis. Another study by Stevens et al. [141] reported that the DM intake and contents of minerals such as calcium, copper, phosphorus, and zinc were higher in control (no hemp meal) diets in comparison to a hemp meal diet. However, there are no studies that have been conducted on the inclusion of cannabis meal in the diet of Dorper sheep in South Africa, future research must be done on Cannabis inclusion in the Dorper sheep diet, and the effects of fermentation on the growth performance of Dorper sheep. Additionally, future research should include adequate levels of Cannabis inclusion in the diet of sheep, and the use of other botanical components of the plant, as most studies are performed on seeds and inflorescence. Furthermore, more studies should be conducted on the fermentation methods, which could aid in eliminating the anti-nutritional factors, thereby increasing the bioavailability of nutrients for ruminants.

## 4. Conclusions and Recommendations

Historical limitations such as legislation have played a significant role in the delay of Cannabis research and development. Notably, Cannabis is a high-value crop due to its excellent nutritional values, and it is also known for its various metabolites, with other metabolites yet to be discovered. Several studies have focused on the nutritional and anti-nutritional factors found in the flowers of Cannabis; however, fewer studies have investigated the nutritional and anti-nutritional factors of other botanical parts of Cannabis plants, such as leaves, stems, and roots, which call for future research. In addition, anti-nutritional factors reduce the bioavailability of nutrients in ruminant feed, and future studies should be conducted on using fermentation as a measure to reduce nutrient loss in ruminant feed. Moreover, there is a need to investigate the metabolites that may be found in the newly developed Cannabis varieties. Alternative fodder resources are continuously being evaluated to advance agricultural production efficiency and profitability. However, Cannabis has potential for use as a high-protein feed source; therefore, understanding the inclusion of *Cannabis sativa* as a supplement for Dorper sheep and its effects on the growth rate require further research, since such studies have not been conducted, and there is no available literature/information in the scientific sources.

## Figures and Tables

**Table 1:** Nutritional compositions of Cannabis seeds, stems, and leaves versus lucerne.

Feed Ingredient	DM (g/kg)	CP	CF	NDF	ADF	ASH	References
Seed	912	246	327	297	213	-	[75]
Stem	-	172	231	-	-	68	[76]
Leaves	887	314	152	471	310	305	[23]
Lucerne	870	198	16	417	333	119	[23]

DM, dry matter; CP, crude protein; CF, crude fiber; NDF, neutral detergent fiber; ADF, acid detergent fiber. ASH represents the total mineral content present in the feed.

## Data Availability

The data presented in this study are available in article.
